# Utilization of Recombinant Baculovirus Expression System to Produce the RBD Domain of SARS-CoV-2 Spike Protein

**DOI:** 10.3390/pathogens11060672

**Published:** 2022-06-10

**Authors:** Youpeng Fan, Junhong Wei, Wei Wang, Chunfeng Li, Guoqing Pan, Timothy Keiffer, Jialing Bao, Zeyang Zhou

**Affiliations:** 1 State Key Laboratory of Silkworm Genome Biology, Southwest University, Chongqing 400715, China; fyp0802@email.swu.edu.cn (Y.F.); weijunhong@swu.edu.cn (J.W.); ww990118@email.swu.edu.cn (W.W.); licf@swu.edu.cn (C.L.); gqpan@swu.edu.cn (G.P.); zyzhou@swu.edu.cn (Z.Z.); 2 Chongqing Key Laboratory of Microsporidia Infection and Control, Southwest University, Chongqing 400715, China; 3 Department of Microbiology and Immunology, Center for Molecular and Tumor Virology, Feist-Weiller Cancer Center, Louisiana State University, Baton Rouge, LA 71130, USA; timothy.keiffer@lsuhs.edu; 4 College of Life Sciences, Chongqing Normal University, Chongqing 400038, China

**Keywords:** baculovirus, BmNPV, BmCPV, COVID-19 spike protein, RBD

## Abstract

Continuous outbreaks of viral diseases in humans facilitates a need for the rapid development of viral test kits and vaccines. These require expression systems to produce a pure and high yield of target viral proteins. We utilized a baculovirus–silkworm expression system to produce the receptor binding domain (RBD) of the SARS-CoV-2 spike protein. First, we had to develop a strategy for constructing a recombinant baculovirus for RBD expression. For this, the coding region of the *Bombyx mori* cypovirus (BmCPV) polyhedron was assembled with the *Bombyx mori* nuclear polyhedrosis virus (BmNPV) promoter. We demonstrated that the recombinant baculovirus has the ability to form polyhedrons within host silkworm cells. In addition, the encapsulated BVs are able to infect silkworms by ingestion and induce foreign protein expression. In this way, we utilized this novel system to obtain a high yield of the target foreign protein, the RBD of the SARS-CoV-2 S protein. However, the viral infection rate of our recombinant BV needs to be improved. Our study shed light on developing a highly efficient expression system for the production of antigens and subsequent immunoassays and vaccines.

## 1. Introduction

A spike protein (S protein) is a large type I transmembrane protein, and usually functions in receptor attachment and membrane fusion [1]. Upon the outbreak of the coronavirus disease 2019 (COVID-19), researchers identified the importance of the SARS-CoV-2 S protein for the coronavirus entering host cells. In particular, the receptor binding domain (RBD) of the S protein is involved in the interactions with the human angiotensin-converting enzyme-2 (ACE-2) receptor and leads to the invasion of the virus [1,2,3]. Since then, studies about the S protein have boomed; therefore, rapid and efficient production systems for the S protein and its RBD are in high demand. Various cell lines have been used to produce the S protein and RBD, such as HEK293 and HeLa cells [4,5]. In addition, the baculovirus vector expression system with *Bombyx mori* nuclear polyhedrosis virus (BmNPV) and the insect cell line Sf9 have been used to produce the SARS-CoV-2 S protein [6]. Coronaviruses including SARS-CoV-2 do not mutate as fast as other RNA viruses due to the NSP14 exonuclease [7,8], yet the spike proteins of the coronaviruses undergo rapid evolution by point mutations and recombination [9,10,11]. As a result, new variants such as the Omicron variant can escape 26 out of 29 monoclonal antibodies that target the spike protein’s receptor binding motif [12]. Therefore, we desperately need to optimize the expression methods in order to rapidly obtain rapidly large amounts of the spike RBD to quickly adapt to any emergent and highly mutated SARS-CoV-2 variants.

Baculovirus gene expression vectors have become an important eukaryotic expression system because the potential level of production of the foreign protein is 20% or more of the total protein of the infected cell. BmNPV is a widely used tool for foreign gene expression and protein production [13,14]. In our previous study, we have established a novel recombinant baculovirus expression system. In that system, we assembled *Bombyx mori* cypovirus (BmCPV) polyhedrin cDNA into BmNPV, and verified that the recombinant virus expression system is feasible and the expression levels of foreign cells have been upregulated.

BmCPV is a non-enveloped dsRNA virus, which specifically infects the midgut epithelium of the host [15]. Mori et al., constructed a recombinant virus expression system by introducing the cDNA segments of BmCPV into recombinant viruses [16]. The ability of the recombinant virus to produce large and inclusion body-like structures were then verified in infected Sf21 and BmN cells. More interestingly, researchers have tried to generate recombinant proteins by infecting producer cells with two baculoviruses simultaneously [17]. Thus, it is of great interest to know whether we can combine the polyhedrin of BmCPV with element(s) of BmNPV and thus generate higher expression levels of foreign proteins in host insect cells.

Here in this study, we generated a novel recombinant baculovirus expression system by combining the BmNPV P10 promotor with the cDNA encoding polyhedrin of BmCPV. This recombinant baculovirus expression system was then utilized to produce the receptor binding domain (RBD) from the spike protein of SARS-CoV-2. The results indicated that the expressed RBD section folded correctly, and the protein yield from host cell line BmE is high. Our study is the first to report the expression of the RBD of S protein of SARS-CoV-2 using a novel recombinant baculovirus system.

## 2. Materials and Methods

### 2.1. Recombinant Virus Construction and Purification

Recombinant proteins were produced using the Bac-to-Bac baculovirus/insect cell expression system (Invitrogen, Carlsbad, CA, USA). Viral genes (p10-poly, E2, CAP) were inserted into the baculovirus vectors pFastbac-Dual using specific primers (Table 1); the sequence of SARS CoVID2 RBD was also inserted into baculovirus vector using specific primers (Table 1). The target sequences of Tobacco Etch virus (TEV) protease, His-tag, and Strep-tag were inserted into the RBD sequence on its C-terminal end. We adopted from studies of recombinant baculovirus [18,19], and developed our novel recombinant baculovirus expression system. The expression of these genes are controlled by the BmNPV polyhedrin (PH) promoter for high-level expression in insect cells, with the expression of BmCPV polyhedrin controlled by the BmNPV P10 promoter. Our cloned gene was introduced into BmNPV bacmid (strain Qd04), and recombinant bacmid was extracted from cultured *E. coli* DH10Bac (Thermo Fisher Scientific, Waltham, MA, USA). The indicated bacmids were transfected into BmE cells according to the manufacturer’s instructions (Bac-to-Bac baculovirus expression system, Thermo Fisher Scientific, Waltham, MA, USA). The baculoviruses (BVs) were purified with a two-step purification protocol first by pelleting lysates via ultra-centrifugation at 200,000× *g* for 2 h at 4 °C (Sorvall WX80+, Thermo Fisher Scientific, Waltham, MA, USA), then running supernatants through a 25% sucrose cushion, and finally resuspending BV pellet(s) in 200 µL PBS.

### 2.2. Cell Culture

*Bombyx mori* cell line BmE-SWU cells were maintained at 28 °C in Grace’s medium (Thermo Fisher Scientific, MA, USA), supplemented with 10% (*v*/*v*) fetal bovine serum (FBS) (Thermo Fisher Scientific, Waltham, MA, USA), and 1%(*v*/*v*) penicillin–streptomycin.

### 2.3. Western Blotting

Western blotting was performed using standard techniques. In brief, cells were lysed on ice using lysis buffer (Beyotime, Shanghai, China) containing 2% Triton X-100 and protease inhibitor cocktail (MCE, USA). Samples were then incubated for 30 min at 4 °C with shaking and centrifuged (10,000 rpm, 15 min, 4 °C), with the supernatants collected. The protein concentrations of supernatants were then equalized and incubated for 10 min in loading buffer (Beyotime). The proteins were separated through electrophoresis, transferred onto polyvinylidene difluoride (PVDF) membranes (Invitrogen), followed by blocking of the membranes for 60 min at 37 °C with 5% nonfat dry milk in a mixture of 0.05 M TBS and 0.05% Tween-20. The membranes were incubated overnight at 4 °C in solution containing mouse anti-His (Sigma, Burghausen, Germany) at a concentration of 1:5000. Subsequently, blots were incubated with an appropriate horseradish peroxidase (HRP)-conjugated secondary antibody (Sigma, Burghausen, Germany), at a dilution of 1:2000 for 1 h at RT. Protein expression was detected with ECL reagents (Thermo Fisher) using the ChemiDoc MP imaging system (BioRad, Hercules, CA, USA), and quantified by densitometry using Image Lab software (BioRad).

### 2.4. Observation of Polyhedrin

The P1 generation BVs were also purified with a two-step purification protocol first by pelleting lysates via ultra-centrifugation at 200,000× *g* for 2 h at 4 °C, then running supernatants through a 25% sucrose cushion, and finally resuspending BV pellet(s) in 200 µL PBS. Purified viruses were observed and quantified using microscopy, 100× objective.

## 3. Results

### 3.1. Construction of Recombinant Baculovirus

**Figure 1:**
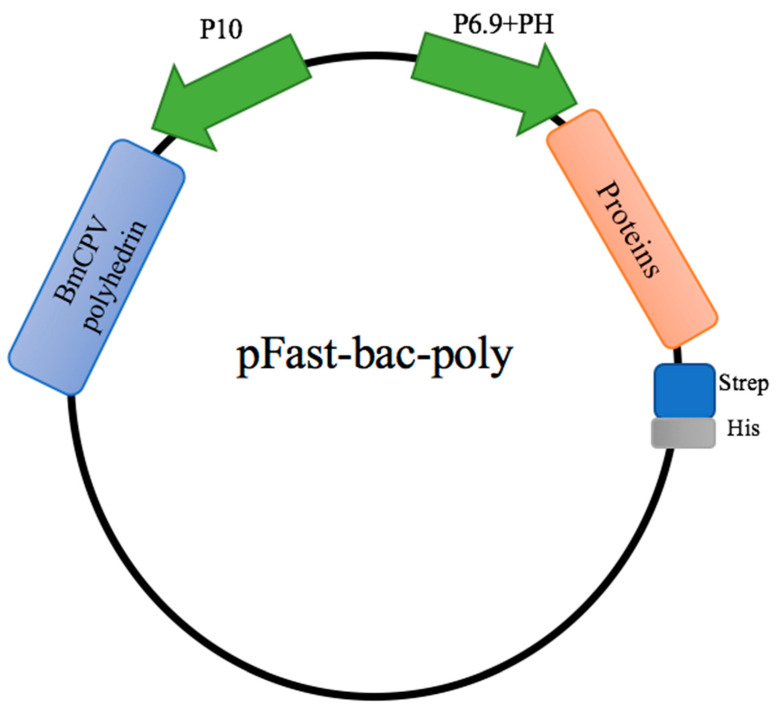
Schematic diagram of pFastbac-Dual vector construction. Viral genes were inserted into the baculovirus vectors pFastbac-Dual, under control of the BmNPV polyhedrin (PH) promoter for high-level expression in insect cells. The expression of BmCPV polyhedrin was controlled by the BmNPV P10 promoter.

For the production of candidate constructs for viral protein production, it is desirable to use a eukaryote system rather than a prokaryotic expression system. Therefore, we applied the BmNPV expression system in BmE cells. We first constructed the pFastbac-Dual plasmid loaded with exogenous protein and BmCPV polyhedron (Figure 1).

### 3.2. Verification of Polyhedron Formation

**Figure 2:**
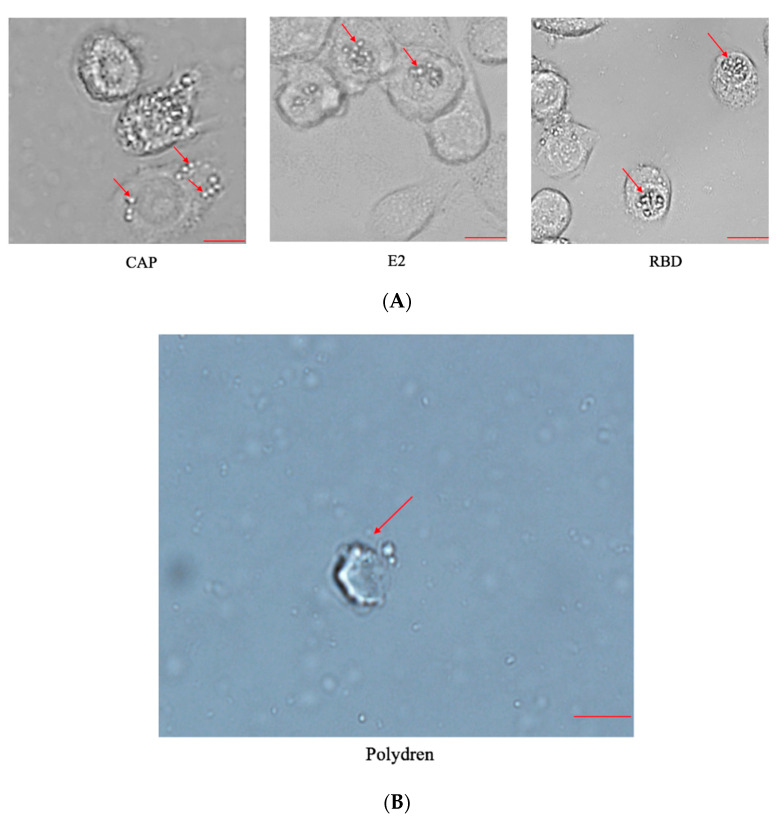
Polyhedron formation. (**A**) Microscopic observation of BmE cells transfected with recombinant bacmid for 4 days. Arrows pointed to signs of polyhedron formation. Scale bar = 10 μm. (**B**) BVs were purified by sucrose cushion and observed under 100× microscope. Arrow points to hexagonal polyhedron structure. Scale bar = 1 μm.

The purpose of expressing the BmCPV polyhedron is to encapsulate BVs so they can infect individual silkworms via ingestion We constructed recombinant BmNPV bacmid as described and then transfected recombinant bacmids, containing the coding sequences of either E2 of African swine fever virus, CAP of porcine parvovirus II, or the RBD of the S protein, into BmE cells. We used bacmids containing E2 or CAP as controls. We observed obvious signs of polyhedron formation 4 days post transfection via microscopy (Figure 2A). These viral particles were then purified as stated previously and we observed that our viral particles were in the conformation of hexagonal polyhedron structures, as seen under a 100× microscope (Figure 2B).

### 3.3. Expression of RBD Domain of S Protein

**Figure 3:**
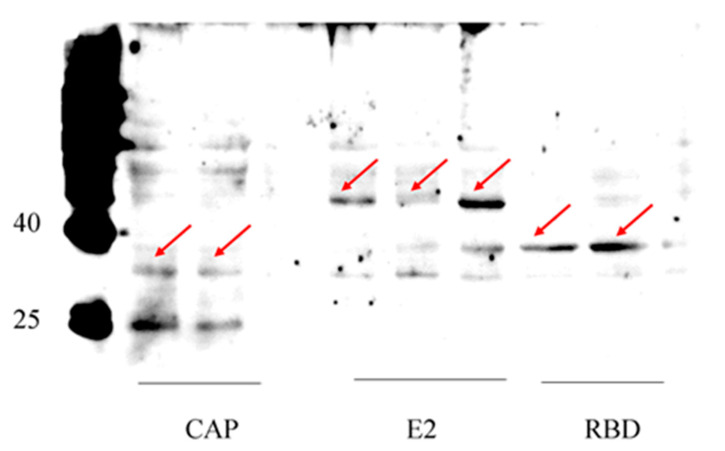
Expression of target proteins detected by Western blotting using anti-his antibody. All proteins were shown at their expected size: RBD domain of S protein (31 kDa), CAP (28 kDa), E2 (26 kDa as monomer, and may form dimer). Arrows pointed to the proteins bands at expected size.

Next, we used the anti-His antibody to detect the expression of viral proteins by Western blotting. Our blots showed that the RBDs of the S protein were successfully expressed by this recombinant baculovirus system. The control proteins E2 of African swine fever virus and CAP of porcine parvovirus II were also correctly expressed (Figure 3). The results showed that our recombinant baculovirus expression system is efficient and versatile for the expression of various kinds of exogenous proteins.

### 3.4. Higher Yield of Protein by Recombinant Baculovirus

To illustrate that the recombinant baculovirus expression system can give a higher yield, we performed a Western blot assay and ELISA assay to compare and quantify the protein yields of the original and recombinant baculovirus systems.

**Figure 4:**
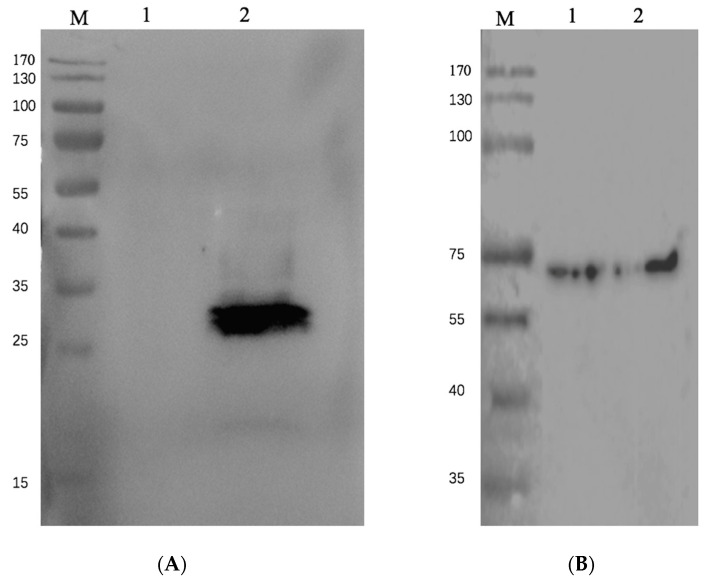
Expression of S protein RBD. (**A**) Western blotting using anti-his antibody. Lane 1 is RBD expressed by pFast-bac expression system, pFast-bac-EGFP-RBD, expressed in BmE cells. Cells were lysed and 10 μl of cell lysate was loaded on gel; Lane 2 is RBD expressed by current recombinant baculovirus system pFast-bac-poly-RBD, expressed in BmE cells. Cells were lysed and 10 μl of cell lysate was loaded on gel. (**B**) Tubulin as internal control, detected by anti-β-tubulin antibody.

**Figure 5:**
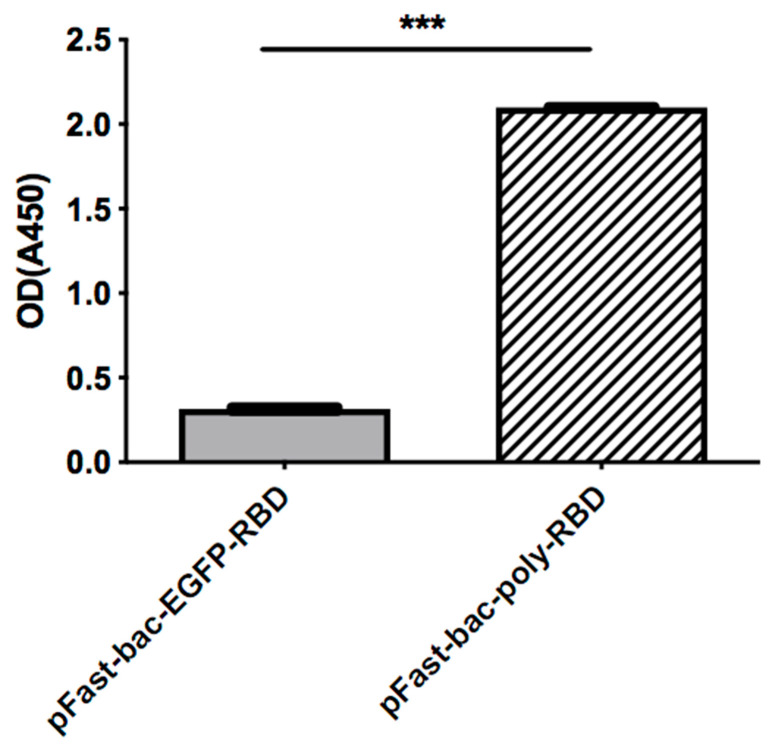
ELISA assay. The expression levels of RBD are compared by ELISA assay, with coated anti-His antibody. The OD450 was measured to illustrate protein quantifications. (*** = *p* < 0.0001).

As shown in Figure 4, the recombinant baculovirus system expressed much more RBD. As quantified by ELISA (Figure 5), the yield of the recombinant baculovirus system is significantly higher than the original system.

## 4. Discussion

The efficiency of recombinant protein production in eukaryotic cells can always stand to be improved, and since the current baculovirus–silkworm expression system is widely used, we wanted to test the effectiveness of this system in producing the SARS-CoV-2 S protein RBD [14,20,21]. In our study, we constructed a recombinant baculovirus in order to obtain the rapid and efficient protein production of RBD. The BmCPV polyhedrin is assembled together with and controlled by the BmNPV P10 promoter. Our results showed that the recombinant baculovirus is able to form the polyhedron, infect host cells, and produce foreign protein with high efficiency.

The RBD domain is an accessible region of the SARS-CoV-2 S protein, thus it has pivotal roles in spike protein-induced viral attachment, fusion, and entry [22,23,24]. The ectodomain characteristic of the RBD on the S protein makes it reasonable to speculate the RBD is more prone to be expressed in a soluble form when exogenously produced. That might explain the high efficiency and yield of the RBD using our newly developed baculovirus expression system. More modifications and improvements might be needed on our vector if we wish to expand using this recombinant virus for broader applications, such as expressing more hydrophobic, large, and insoluble proteins.

Another important improvement of this new recombinant expression system is that it can be used to screen other host cell lines to find the most amendable cell line(s) for protein expression. We now have tried *B. mori* BmE cells and BmN cells, and found our recombinant virus performed better using BmE cells. Yet, we cannot neglect the usage of other insect cells line, such as Sf9 and Sf21, which are all widely applied in the baculovirus–insect cell expression system, to express exogenous proteins [25,26,27]. It will be necessary to test our virus in these various cell lines and find the best conditions for producing exogenous proteins.

In summary, we have established a novel way of assembling BmNPV with elements of BmCPV. This recombinant baculovirus is able to form a polyhedron and produce exogenous protein in BmE cells; in our case, we produced an important, currently-relevant protein in the RBD domain of the S protein of SARS-Covid-2. We expect to utilize this system for producing various viral proteins and thus provide a useful tool for immune assays and vaccine development.

## Figures and Tables

**Table 1:** Sequences of primers used in this study.

Primer Name	Primer Sequence
>F-p10-poly	CCGCTCGAGATGGCAGACGTAGCAGGAACA
>R-p10-poly	CGGGGTACCCTGACGGTTACTCAGAGCTAC
>F-E2	CTTTACTTCCAGGGAGGATCCATGGTTTCCAGGTTTTTAATA
>R-E2	ATGGTGATGGTGGTGAGCTTCTCATCATCCTCCTCTTC
>F-CAP	CTTTACTTCCAGGGAGGATCCATGACGTATCCAAGGAGGC
>R-CAP	ATGGTGATGGTGGTGAGCTTAGGGTTAAGTGGGGGGTCTTT
>F-RBD	CTTTACTTCCAGGGAGGATCCATGACTGAATCTATCGTGAGA
>R-RBD	ATGGTGATGGTGGTGAAGCTTTTCCAGAGTTTGTGGGTCTT

## Data Availability

The data presented are within this manuscript, and available upon request if more details needed.
